# Secondary or New Formation of Teeth

**Published:** 1887-06

**Authors:** Wm. A. Powers


### Secondary or New Formations of Teeth.

BY WM. A. POWERS.

It seems best to study first the hard formations within the pulp chamber.

Prominent among these is secondary dentine, a new growth of dentine more or less regular in formation, excited by abrasion, decay, or mechanical injury by which the dentinal fibres are subjected to irritation at their distal ends. It is deposited upon the walls of the pulp chamber and reduces its size. It is distinguished from the normal dentine by its different appearance. The tubuli usually change their direction somewhat, and there is a diminution in numbers. This departure from the normal structure varies greatly in different cases; sometimes the diminution in numbers will be the only change from normal. Again the change is so slight as to be almost imperceptible; this condition, however, is not frequent. Usually there is quite a difference in the color of the new formation compared with the normal dentine.

The deposit is said to be more extensive in the incisors and cuspids than in the molars. In the molars the formation is confined almost entirely to the bulb of the pulp, extending into the root portion very little. The deposit is quite uniform on the walls of the chamber. A remarkable fact concerning this growth is that when there is considerable wear of the teeth generally those that have escaped abrasion will have about the same amount of secondary dentine as those that have been abraded.

When the deposit is excited by caries there are features about it which distinguish it from that excited by abrasion. As has been seen the growth caused by abrasion was quite generally deposited upon the walls, while that resulting from caries is usually confined to a part of the pulp chamber immediately opposite the carious portion. It is thought by some authors that any considerable growth of secondary dentine is followed by exhaustion and finally death of the pulp.

These formations are mostly found in persons of middle age, occasionally in young persons, and they also occur in cases where the irritation has been long continued. Oftener found in teeth having many cavities than those comparatively free from decay.

Another important formation is osteo-dentine. This is a term applied to a hard substance partaking both of the nature of bone and dentine, but more analogous to the former than the latter, deposited on the inside of dentine usually after the age of twenty. The entire pulp is sometimes converted into this substance. This term excludes all formations in which are not found bone corpuscles. In human teeth these are very rare, but are frequently met with in the teeth of animals, especially large animals. They present the general characteristics of cementum and are found in the root canal either attached to the dentine or slightly separated from it.

Dentinal tumors in the pulp chamber are forms of secondary dentine which are sometimes seen. They are masses of calcific material which are attached to the wall of the pulp chamber by a stem or pedicle. The tubules seemingly have no definite direction but curl, curve, and branch ending in blind extremities. The causes which produce the growth are similar to those forming secondary dentine generally. Why the growth takes this peculiar form is unknown.

Under the head of Calification of Pulp Tissues we have infiltration of the pulp tissues with lime salts. As a rule the form of the tissue elements is not preserved sufficiently well to enable us to identify it as pulp tissue, although there is occasionally such a case. They have generally a smooth surface and quite regular outline. Accompanying tissue calcification we have degeneration of the remaining pulp tissue. Sometimes the calcification involves the entire pulp; the new formation perfectly fitting the walls of the pulp chamber. These masses are evidently formed very slowly and may begin at any part of the pulp.

Cylindrical calcification may be considered as a sub-division of the above. It is a peculiar form of interstitial calcification of the pulp occurring only in the root canal. This occurs in patients of all ages, but perhaps more frequently in middle aged people. In the early stages the calcific material is found in the tissues in extremely small spindle-shaped, or cylindrical masses. A pulp having these formations will be somewhat stiffened so that it will retain its shape after being bent. As the calcification progresses the cylinders become attached to each other, but preserve their jointed appearance, being connected by fibres—what forces are operating upon, or what peculiar condition of the pulp is necessary, to produce this form is unknown.

### PULP NODULES.

These are irregular nodulated masses frequently seen. They may be found in any part of the pulp-tissue, but oftener occur in the coronal portion, or near the junction of this with the root portion. Irregular in form and nodulated, very hard, and composed of the same material as the dentine, but structurally different, they are not calcifications of the pulp tissue, but deposits in the inter-spaces making room for themselves by crowding or pushing the tissues aside. These should not be confounded with calcific degeneration of the tissue-fibres which become infused with lime-salts. Both are irregular bodies and are sometimes found together. The nodules found in the root portion of the canal are smoother than those in the coronal, and more prone to contain calcified tissues. They are more frequently in the teeth of middle aged and old people than in the young. They appear to give no trouble unless by their size they obstruct the circulation, or impinge upon nerve fibres.

### CALCO-GLOBULAR.

This is associated with pulp-nodules and has the same form of elements, but is soft while the nodule is hard. It is usually found near the point of exposure many times lying directly beneath the layer of odontoblasts but not infrequently found deeper. It is found in irregular masses sometimes of considerable size. The artificial formation of these bodies seems to require a solution of albumen, salts of lime, and carbonic acid. In varicose veins there is a super-saturation of the blood with carbonic acid and as these bodies are only found where there is inflammation it is possible that the formation of these bodies in the dental pulp is dependent upon a congested condition of the vessels of the pulp.

### EXOSTOSIS.

The only part of a tooth subject to this formation is the root, and it usually commences at or near the apex, extending from that point upwards towards the crown covering more or less of the external surface. Sometimes it begins on the side and quite a large deposit of osseous matter takes place; again the bony substance is regularly deposited over nearly the whole surface of the root. It is generally dense in structure, of a slight yellowish hue, and semi-transparent.

Usually it is a mere increase of the normal tissue without vessels. Sometimes, however, blood-vessels are involved in the growth. It may be cancellated and porous. We may consider it as layers of tooth-bone formed by hypertrophic action, the laminæ quite marked, while the lacunæ are large and abundant.

